# LLM‐Guided Parameter Optimization for Mechanistic CHO Cell Bioreactor Models

**DOI:** 10.1002/bit.70300

**Published:** 2026-07-01

**Authors:** Han Bit Kim, Janghan Lee, Seo‐Yeon Kim, Jihoon Kim, Moo Sun Hong

**Affiliations:** ^1^ Department of Chemical and Biological Engineering Seoul National University Seoul Republic of Korea; ^2^ New Drug Development Center, Osong Medical Innovation Foundation Cheongju Republic of Korea; ^3^ College of Pharmacy Chungbuk National University Cheongju Republic of Korea

**Keywords:** bioreactor modeling, CHO cells, large language models (LLMs), mechanistic modeling, parameter estimation

## Abstract

Mechanistic models of Chinese Hamster Ovary (CHO) cell cultures are essential for efficient bioprocess development and control, yet parameter estimation remains a critical challenge in model calibration. As model complexity increases, the number of parameters grows and their values become highly system‐specific due to biological variability across cell lines and operating conditions. Consequently, estimation requires broad search spaces, leading to inefficient exploration and increased risk of entrapment in local minima during numerical optimization. To address this, a structure‐aware optimization framework is proposed that integrates large language model (LLM) reasoning into the parameter estimation loop. The LLM analyzes governing equations and trajectory discrepancies to recommend directional adjustments for parameter boundaries. These insights are combined with correlation‐based scaling to adaptively refine the search space, which is explored using stochastic global search methods (Latin hypercube sampling [LHS] and Covariance matrix adaptation evolutionary strategy [CMA‐ES]). Applied to fed‐batch CHO cell culture data, the framework escapes local minima and resolves complex, nonlinear metabolite dynamics, including lactate and ammonium shifts. Statistical validation across multiple batches and random seeds shows consistent reduction in prediction error and improved optimization robustness. This study demonstrates the potential of integrating structure‐aware LLM guidance with numerical optimization to improve the reliability and efficiency of bioprocess modeling.

## 1 Introduction

Monoclonal antibodies (mAbs) remain a dominant class of biopharmaceuticals, with Chinese Hamster Ovary (CHO) cells serving as the industry‐standard production platform due to their robust culture adaptability and human‐compatible glycosylation profiles (Schulz et al. 2026). To support efficient process development and control, mechanistic models have become increasingly important, providing fundamental insights into cellular metabolism and enabling model‐based design, monitoring, and scale‐up (Ranpura et al. 2026). These models also form the core of emerging digital twin frameworks in biomanufacturing (Park et al. 2024).

Despite their advantages, reliable parameter estimation remains a major bottleneck in the practical deployment of mechanistic CHO bioreactor models (Babel et al. 2025). As model complexity increases, the number of parameters grows substantially, and their values become highly system‐specific due to biological variability across cell lines and operating conditions (Lu et al. 2024). This results in poorly defined parameter bounds and high‐dimensional search spaces that are difficult to explore efficiently.

A variety of optimization methods, including gradient‐based, stochastic, and evolutionary approaches, have been widely applied to parameter estimation. However, in high‐dimensional bioprocess systems, their performance is often limited by the structure of the parameter search space. Poorly defined and overly broad parameter bounds lead to inefficient exploration, slow convergence, and frequent entrapment in local minima, fundamentally constraining optimization performance across methods (Villaverde et al. 2019; Li and Vu 2013). Consequently, unguided numerical optimization alone is often insufficient to reliably identify physically meaningful parameter sets.

To address this limitation, this study introduces a mechanistic structure‐aware strategy for adaptive parameter space refinement, in which a large language model (LLM) is embedded within the optimization loop. Rather than relying solely on numerical search heuristics, the LLM interprets the governing equations, functional roles of parameters, and discrepancies between model predictions and experimental data to guide the search process. This approach builds on recent advances in LLM‐based mathematical reasoning and scientific modeling (Taskin et al. 2026; Wang et al. 2026; Du et al. 2024; Luo et al. 2025; Androulakis et al. 2025). Based on this structural understanding, the LLM identifies influential parameters and recommends directional adjustments to their search boundaries. These updates are further integrated with correlation‐based scaling to account for parameter interdependencies, enabling coordinated refinement of the search space.

The resulting framework combines LLM‐guided boundary adaptation with a two‐stage numerical optimization strategy, consisting of stochastic global search (Latin hypercube sampling [LHS] and Covariance matrix adaptation evolutionary strategy [CMA‐ES]) followed by gradient‐based local refinement (L‐BFGS‐B). By reshaping the parameter space toward regions that are both physically plausible and optimization‐relevant, the proposed approach improves convergence efficiency and robustness.

The proposed approach is demonstrated using fed‐batch CHO cell culture data, where it consistently enhances convergence behavior, reduces optimization loss, and enables escape from local minima. These results highlight the potential of integrating mechanistic structure‐aware reasoning with numerical optimization to improve the practical usability of mechanistic models in bioprocess applications.

## 2 Materials and Methods

### 2.1 Experimental Methods

mAb‐producing CHO cells were cultured in a 14‐day fed‐batch process using an Ambr 15 automated microbioreactor system (Sartorius) with an initial working volume of 15 mL. Cultures were inoculated at $0.3\times 10^{6}$ cells/mL, with dissolved oxygen (DO) maintained at 40%. Bolus feeding (5% v/v) was performed on Days 2.6, 4.6, 6.6, 8.6, 10.5, and 12.6, while glucose was continuously supplemented to maintain a concentration of ≥ 3 g/L. Culture dynamics were monitored by sampling on Days 0.6, 2.6, 4.6, 5.6, 6.6, 7.6, 8.6, 10.5, 12.6, and 13.5. Eight parallel batches were cultivated under varying pH and temperature strategies, with pH setpoints of 6.8, 7.0, 7.2, and 7.4. Four batches were maintained at a constant $37^{\circ }$C, while the remaining four underwent a temperature downshift to $35^{\circ }$C on Day 5. All cultivations were terminated on Day 14, and the supernatant was collected via centrifugation for analysis.

### 2.2 Model Equations

The mechanistic CHO bioreactor model employed in this study is formulated as a system of ordinary differential equations (ODEs). The baseline mass balance equations and kinetic rate expressions were adapted from the model proposed by Craven et al. (2013). To capture the specific kinetic characteristics of the cell line, the governing equations were modified in two aspects. First, the formulation for the specific death rate ($\mu_{d}$) was adopted from Xing et al. (2010) to better represent cell death kinetics driven by byproduct toxicity. Second, a specific production term ($k_{\text{Gln},\text{prod}}\frac{K_{\text{Gln},\text{prod}}}{K_{\text{Gln},\text{prod}}+\text{Gln}}$) was incorporated into the glutamine mass balance to explicitly account for glutamine synthesis. The complete set of governing ODEs, kinetic expressions, and state variable definitions is provided in Section S1 of the Supporting Information.

### 2.3 LLM‐Guided Parameter Optimization

This study proposes a novel, integrative optimization pipeline that combines mechanistic, structure‐aware LLM reasoning with numerical search algorithms. Because constant parameters are insufficient to capture the dynamic behavior and metabolic shifts of CHO cells, particularly for lactate and ammonium (Ben Yahia et al. 2017), parameter estimation is conducted in two distinct phases separated at Day 7.6. This phase‐wise formulation mitigates structural underfitting and enables representation of distinct metabolic regimes. As illustrated in Figure 1, within each phase, the framework operates cyclically, starting from an a priori parameter search space and proceeding through a two‐stage numerical optimization process. The initial search boundaries for the parameters are generally defined within a logarithmic range of ±2 (i.e., $10^{-2}$ to $10^{2}$ relative to their predefined default values). However, specific adjustments are made for certain parameters to account for physiological relevance and numerical stability. For instance, based on physiological considerations and literature‐reported ranges, the boundary for the maximum specific growth rate ($\mu_{g,\max}$) is set to [0.2,10]. Furthermore, for the maintenance coefficients $m_{G}$ and $m_{Q}$, which possess inherently small default values of $10^{-6}$, the lower bounds are restricted to $10^{-8}$ to maintain numerical stability during the optimization process. The predefined default values for all parameters are summarized in Table S1 of the Supporting Information. The resulting optimized parameters and model predictions are subsequently analyzed by an LLM agent and a numerical correlation module to adaptively refine and coordinate the search boundaries for the next optimization cycle.

**Figure 1:**
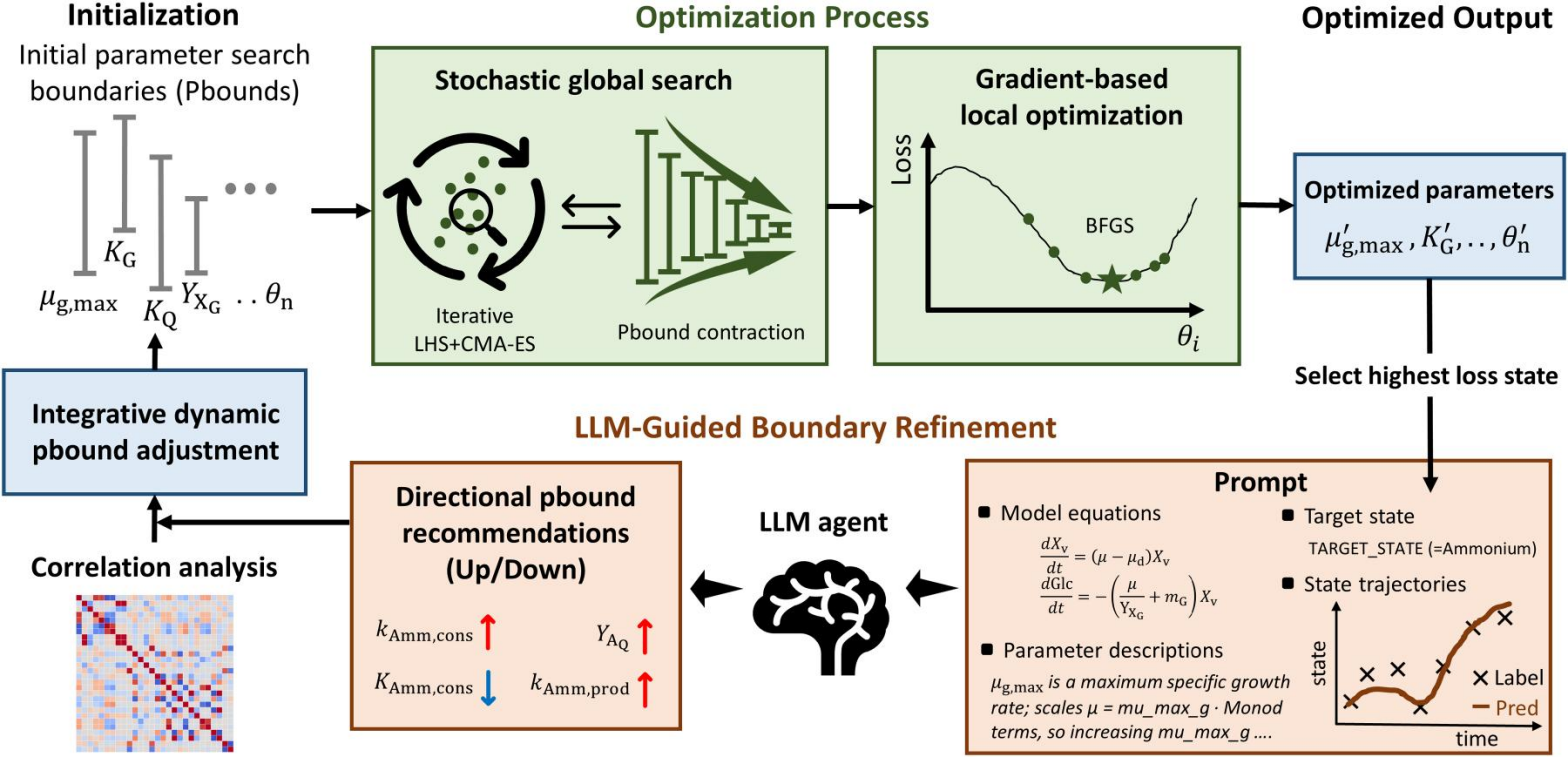
Overall workflow of the proposed LLM‐guided parameter optimization framework for CHO mechanistic models. The pipeline consists of an iterative two‐stage numerical optimization (stochastic global search using LHS and CMA‐ES, followed by gradient‐based local refinement using L‐BFGS‐B) combined with LLM‐guided boundary adaptation. Based on model equations, parameter roles, state trajectory discrepancies, and a specified target state, the LLM provides directional recommendations for multiple relevant parameters. These parameter updates are further coordinated through correlation analysis to iteratively refine the search space.

#### 2.3.1 Optimization Process

The optimization process is conducted in two sequential stages: an iterative stochastic global search followed by a gradient‐based local optimization. First, the stochastic global search systematically explores the parameter space over multiple global iterations ($n_{\;\text{global\_iter}\;}$), using LHS and CMA‐ES optimization steps. Within each global iteration, a fixed number of candidate parameter sets ($n_{\;\text{exp\_per\_iter}\;}=10,000$) are evaluated. These algorithms are applied sequentially: the first half of the computational budget (5000 evaluations) is allocated to LHS for broad exploration of the parameter space. Based on this initial exploration, CMA‐ES is subsequently applied using the remaining 5000 evaluations to adaptively refine the search around promising regions.

The objective loss evaluated during this optimization process was calculated by applying min–max normalization to each state variable using the maximum and minimum values of the experimental data. The mean absolute error (MAE) was then computed for each state. A weighting factor of 7 was applied exclusively to the viable cell density MAE, as $X_{v}$ and the specific growth rate (μ) exert a dominant biological and mathematical influence on the overall ODE system dynamics. The final objective loss was determined by averaging the resulting state‐specific losses.

Crucially, while conventional CMA‐ES employs a population size—defined as the number of candidate evaluations per evolutionary generation—that scales logarithmically with the number of parameters (typically 4+⌊3ln(N)⌋, where N is the parameter dimensionality), this framework employs an intentionally enlarged population size of $0.1\times n_{\;\text{exp\_per\_iter}\;}$. This design maintains high search diversity while limiting the number of evolutionary generations to five per global iteration, effectively preventing premature convergence in high‐dimensional bioprocess models.

Following the hybrid search within each global iteration, the overarching parameter boundaries are dynamically contracted around the top‐performing 20 candidates. This iteratively refined space becomes the updated bounds for the subsequent iteration. The process continues for up to a maximum of $n_{\;\text{global\_iter}\;}=60$, incorporating an early stopping criterion (loss improvement < 10^−4^ over 10 consecutive iterations). Finally, the best parameter set identified ($\theta_{\text{global}}^{*}$) serves as the initial guess for the L‐BFGS‐B local optimization, which is constrained within a ±20% range of $\theta_{\text{global}}^{*}$ to refine the parameter estimates and locate the local optimum, yielding the optimized parameter set ($\theta^{*}$).

To further evaluate the efficacy of the LLM‐guided framework in escaping local minima relative to purely numerical stochastic optimization, an additional comparison was performed using the basin‐hopping algorithm. The basin‐hopping search was initialized with the initial optimized parameter set ($\theta^{*}$) and configured with 10 independent stochastic runs, each performing 10 iterations (totaling 100 hops). This computational budget was chosen to ensure practical feasibility while allowing for much broader stochastic exploration, whereas the proposed LLM‐guided approach requires only a minimal number of targeted updates (specifically, one or two). The L‐BFGS‐B algorithm was employed as the local minimizer, and the temperature parameter was set to 0.2, corresponding to the characteristic scale of the objective loss (~0.1), to allow sufficient stochastic exploration during hopping.

#### 2.3.2 LLM‐Guided Boundary Refinement

Following the numerical optimization, the pipeline evaluates the normalized MAE for each state variable. The state exhibiting the highest normalized error is selected as the target for LLM intervention.

In this framework, gpt‐oss‐120b (OpenAI 2025) was employed as the core LLM agent. This locally deployable open‐source model was selected to avoid external transmission of confidential process data and to ensure long‐term reproducibility. Furthermore, despite having a smaller parameter scale than larger proprietary commercial models, it demonstrated sufficient performance for the proposed optimization task. The structure‐aware LLM inference begins by constructing a structured prompt. The prompt is designed to provide the LLM with both the quantitative state of the current optimized parameters and heuristic rules for parameter adjustment. Specifically, the agent is supplied with dynamic inputs, including the mechanistic governing equations, parameter descriptions, and unnormalized experimental trajectories versus predictions of the target state. Within this framework, the governing ODEs constrain the parameter selection to mechanistically relevant parameters, while discrepancies between the model predictions and experimental trajectories determine which parameters should be adjusted and in which direction. Notably, the parameter descriptions were not manually engineered; rather, they were generated by the LLM in a preliminary step by reasoning through the provided model equations. For instance, the LLM inferred the systemic role of the maximum specific growth rate ($\mu_{g,\max}$), describing it as: “Maximum specific growth rate; scales $\mu =\mu_{g,\max}\cdot \;\text{Monod terms}\;$, so increasing $\mu_{g,\max}$ proportionally boosts $\left(\mu -\mu_{d}\right)\cdot X_{v}$ growth term, accelerating viable cell accumulation and glucose and glutamine consumption.” Furthermore, the agent is explicitly instructed to leverage heuristic rules, such as balancing production and consumption terms for nonlinear, concave/convex trajectories, or isolating dominant kinetic terms for linear trends. The complete architectural framework and the exact raw text of the prompt utilized in this study are detailed in Section S2 of the Supporting Information.

To quantify the interdependencies among the kinetic parameters, a correlation analysis is conducted. A covariance matrix is computed by applying finite‐difference perturbations to the current candidate parameters ($\theta^{*}$), which is subsequently normalized to obtain the numerical correlation matrix.

The outputs from these two analytical components—the qualitative directional guidance from the LLM and the quantitative correlation matrix—are synthesized within the integrative dynamic bound adjustment module to update and coordinate the search space for the next optimization cycle. All updated boundaries are formulated on a logarithmic scale.

To formalize this mechanism, let $\theta_{k}^{*}$ represent the current optimized value of any parameter in the model. The algorithm applies an asymmetric adjustment to the primary parameters explicitly targeted by the LLM to drive the search. Conversely, to preserve the balance of the mechanistic model, the boundaries of all remaining secondary parameters are symmetrically widened based on their mathematical interdependency. Incorporating a minimal numerical buffer ($\epsilon =10^{-5}$) and a fixed logarithmic moving magnitude of 2 for primary updates, the dynamically updated bounds for any parameter $\theta_{k}$ are defined as:

(1) $$\;\text{Bounds}\;\left(\theta_{k}\right)=\left\{\begin{array}{ll} \left[\theta_{k}^{*}\times 10^{-0.1},\theta_{k}^{*}\times 10^{1.9}\right]\; & \;\text{if explicitly targeted by LLM and recommended}\;\;\mathrm{‘up’}\\ \left[\theta_{k}^{*}\times 10^{-1.9},\theta_{k}^{*}\times 10^{0.1}\right]\; & \;\text{if explicitly targeted by LLM and recommended}\;\;\mathrm{‘down’}\\ \left[\theta_{k}^{*}\times 10^{-c_{k,\max}∕2-\epsilon },\theta_{k}^{*}\times 10^{c_{k,\max}∕2+\epsilon }\right]\; & \;\text{if a secondary parameter (not explicitly targeted)}\; \end{array}\right.$$

where $c_{k,\max}$ denotes the maximum absolute correlation coefficient between the secondary parameter $\theta_{k}^{*}$ and the set of LLM‐targeted primary parameters. Ultimately, this integrative update mechanism ensures that the search space is both directed toward physically plausible regions suggested by the LLM and coordinated to respect intrinsic parameter correlations.

## 3 Results and Discussion

### 3.1 Efficacy of Iterative Boundary Contraction in Global Optimization

To establish a robust baseline for the pipeline, the operational logic of the progressive parameter space refinement in the optimization process was first validated. A comparative analysis demonstrated that iterative contraction of parameter boundaries around top‐performing candidates outperforms static wide‐bound searches, improving convergence efficiency in high‐dimensional parameter space (Figure S1a). Furthermore, analysis of the convergence trajectory across iterations revealed a consistent decrease in objective loss accompanied by diminishing marginal improvements, supporting the implementation of an early stopping criterion (Figure S1b). Accordingly, terminating the global search when the objective function fails to exhibit meaningful improvement over 10 consecutive iterations provides an effective balance between computational efficiency and optimization accuracy. This streamlined baseline consistently yields a well‐refined candidate parameter set ($\theta^{*}$), which serves as the basis for subsequent structure‐aware LLM intervention. Details are provided in Section S3 of the Supporting Information.

### 3.2 Mechanistic Analysis of LLM‐Guided Escape From Local Minima

To rigorously evaluate the capability of the proposed structure‐aware LLM reasoning, it was investigated whether LLM‐guided parameter boundary updates could rescue optimization processes trapped in local minima. The numerical optimization was performed under robust conditions ($n_{\;\text{global\_iter}\;}=60$ and $n_{\;\text{exp\_per\_iter}\;}=10,000$ with early stopping) to establish a fully converged baseline. Following this, the LLM agent analyzed the mechanistic structure and trajectory discrepancies of the target states to propose directional boundary updates, after which the optimization was re‐executed to convergence.

The core results demonstrating the algorithm's capability to successfully escape local minima are summarized in Figure 2. It is important to note that while the baseline numerical optimization does not inevitably fail in all instances, it remains highly vulnerable to becoming trapped in suboptimal local minima. As illustrated by the state trajectories of a specific batch where ammonium fitting failed (Figure 2a), and further supported by detailed lactate and ammonium close‐ups from other batches (Figure 2d), the baseline optimization (“Initial opt.”, dashed red line) converged to such local minima. This phenomenon was particularly prominent for ammonium and lactate predictions, as these metabolite trajectories exhibit complex, nonlinear shifts between net consumption and production rates. In the high‐dimensional parameter space, the unguided optimizer often prioritizes fitting dominant macroscopic states (e.g., cell growth) to minimize the overall objective loss, thereby sacrificing fidelity in capturing these intricate metabolic dynamics. When the numerical optimizer becomes trapped in this compromised state, the LLM‐driven mechanistic interpretation of the governing equations enables targeted parameter adjustments that facilitate escape from local minima.

**Figure 2:**
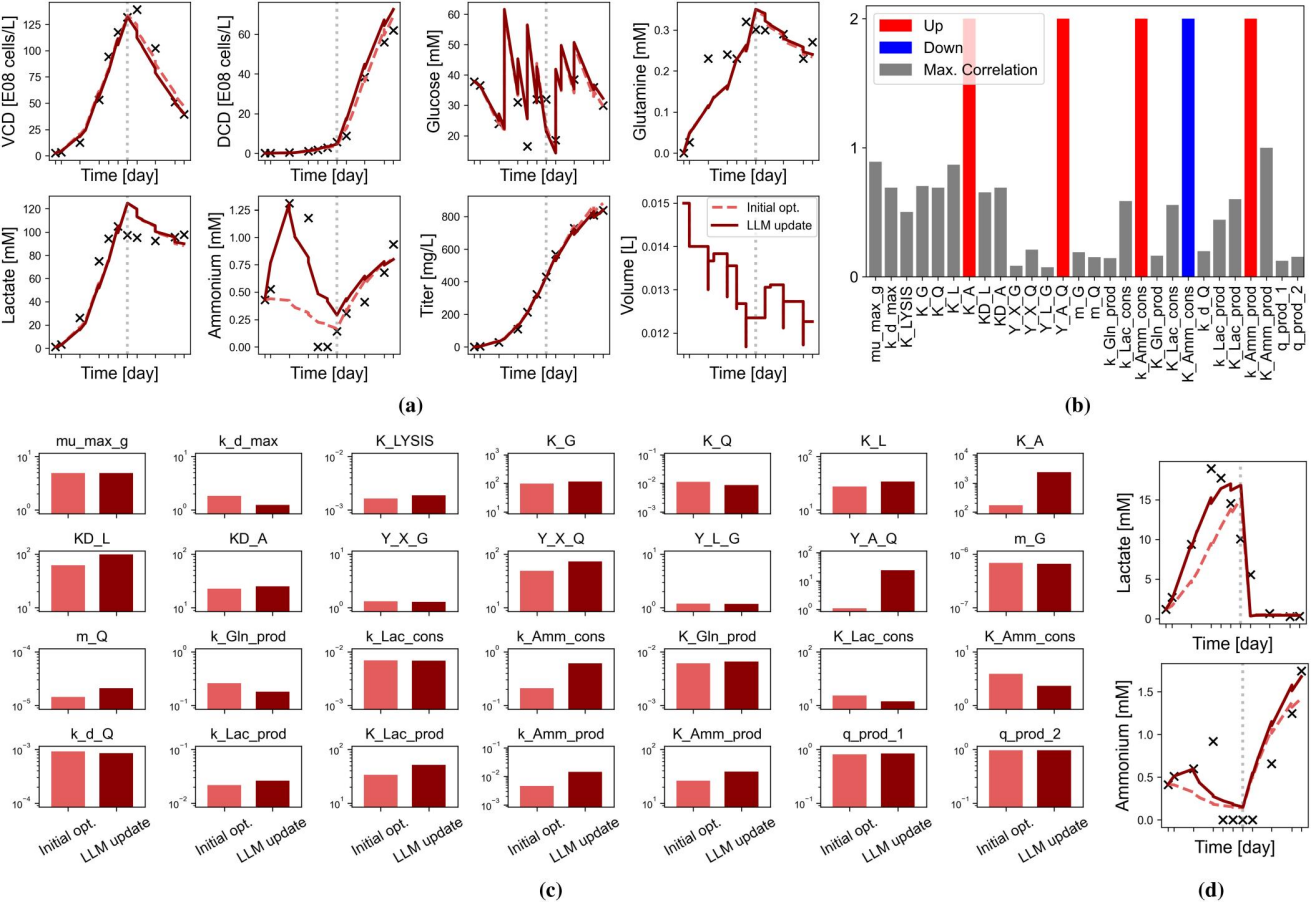
Analysis of LLM‐guided parameter boundary updates for escaping local minima. (a) Improved mechanistic model predictions (solid dark red line) against experimental data (crosses) following a single LLM‐guided update targeting ammonium, compared to a fully converged numerical optimization trapped in a local minimum (dashed red line) for a representative batch among the eight parallel cultures. Time‐series trajectories are shown for key states over 14 days of cultivation, with the vertical dotted gray line indicating the separation of the two distinct parameter estimation phases. (b) Algorithmic formulation of the updated search boundaries for the first phase of this batch. The plot displays the explicit directional recommendations from the LLM (red bars for “up,” blue bars for “down”) alongside the dynamically calculated boundary widths for secondary parameters based on correlation analysis (gray bars). (c) Corresponding shifts in specific optimized parameter values within this first phase (light red bars for initial optimum vs. dark red bars for post‐update optimization). (d) Focused close‐ups for lactate and ammonium from other batches, highlighting the successful capture of complex, nonlinear dynamics (consumption‐production switches) that conventional parameter searches fail to capture.

As outlined in the methodology, the LLM evaluates the system to provide directional guidance for updating the parameter search space (Figure 2b). In this case, correcting the ammonium trajectory requires simultaneous adjustment of both production and consumption fluxes. The reasoning generated by the LLM reflects an attempt to balance these competing fluxes to reproduce the observed dynamics (details are provided in Section S4 of the Supporting Information). The core rationale was as follows:

Ammonium dynamics are governed by production […] and consumption […] The measured ammonium profile shows a pronounced early peak that the model under‐predicts, followed by a rapid decline to near‐zero that the model over‐predicts. To raise the early peak we increase the overall production capacity (raise $Y_{\;\text{A/Q}\;}$, raise $k_{\text{Amm},\text{prod}}$, raise $K_{A}$ to lessen ammonium inhibition of growth and thus boost $r_{\text{gln},\text{cons}}$). To sharpen the later decline we increase the consumption capacity (raise $k_{\text{Amm},\text{cons}}$) and make the consumption term less saturated (lower $K_{\text{Amm},\text{cons}}$). […]

This rationale demonstrates that the LLM correctly identified the key parameters governing ammonium production and consumption through a coupled selection process based on the ODE structural constraints and the experimental trajectory discrepancies. Specifically, the LLM first restricts the candidate parameter set based on the governing ODE terms (e.g., “Ammonium dynamics are governed by production… and consumption…”) and subsequently determines the directional parameter updates required to reproduce the observed trajectory discrepancies (e.g., “The measured ammonium profile shows a pronounced early peak that the model under‐predicts…”). In particular, it targeted $Y_{\;\text{A/Q}\;},k_{\text{Amm},\text{cons}},k_{\text{Amm},\text{prod}}$, and $K_{\text{Amm},\text{cons}}$ to address the transient peak and subsequent clearance. However, its justification for $K_{A}$ reveals a limitation in causal interpretation. The LLM inferred that increasing $K_{A}$ would reduce growth inhibition and enhance glutamine consumption. This interpretation is not mechanistically consistent, as the viable and dead cell density trajectories were already well‐captured, leaving little room for changes in the specific growth rate. Consequently, increasing $K_{A}$ does not significantly change growth dynamics. Instead, it reduces the inhibitory effect of ammonium fluctuations on cell growth and stabilizes the growth profile.

Figure 2c shows the resulting shifts in optimized parameter values. While the incorrect reasoning did not degrade performance in this instance, it highlights the risk of text‐based causal misinterpretation. To mitigate this risk and avoid over‐constraining the search space, a numerical safety buffer (e.g., a $10^{-0.1}$ margin in the opposite direction) is applied to all LLM‐recommended updates. This design allows the numerical optimizer to recover from potentially inaccurate guidance and preserves the robustness of the overall optimization process.

Furthermore, while LLM‐guided updates are constrained to at most five highly influential parameters, the algorithm intrinsically prevents omitting other critical variables. If an unselected parameter must undergo a substantial shift to fully escape the local minimum, it typically exhibits a strong mathematical interdependency with the LLM‐targeted set. Consequently, the correlation‐driven boundary adjustment mechanism assigns a proportionally widened search space (indicated by the gray bars in Figure 2b) to these correlated secondary parameters, ensuring the subsequent numerical optimizer has ample flexibility to naturally discover their appropriate values. It is important to acknowledge that shifting the initial search boundaries in a specific direction does not mathematically force the subsequent optimizer to strictly settle at a proportionally higher or lower value. However, this combination of explicit directional guidance and correlation‐based boundary expansion effectively perturbs the optimization landscape, guiding the optimization process toward a more promising and physically plausible parameter domain.

### 3.3 Statistical Validation of LLM‐Guided Parameter Optimization

While the previous section demonstrated the mechanistic capability of the LLM to rescue the optimization process in a representative scenario, a rigorous statistical evaluation is required to ensure that the observed loss reductions arise from structure‐informed guidance rather than stochastic variability. Because the global search algorithm is inherently stochastic, the improvements observed in Section 3.2 may arise from a favorable stochastic search trajectory, rather than from the LLM‐guided boundary updates. To assess the consistency of this improvement, the analysis was extended across all eight bioreactor batches, each of which was calibrated independently to account for its specific cultivation condition and metabolic behavior. For each batch, both the initial wide‐bound optimization and the subsequent LLM‐guided optimization were evaluated across five independent random seeds, enabling direct statistical comparison beyond stochastic variability in the optimization process.

Figure 3 presents the aggregated loss distributions evaluated across five independent random seeds for each of the eight individual batches. The reductions in loss are characterized by a systematic downward shift in the distributions across runs, reflecting a consistent reshaping of the optimization landscape toward lower‐error regions. This suggests that the improvements are not attributable solely to stochastic search variability, but instead arise from the structure‐informed boundary updates introduced by the LLM. To further distinguish the effect of the proposed framework from purely numerical stochastic optimization approaches, a comparison was performed against the basin‐hopping algorithm, a widely used stochastic global optimization method for mitigating local minima. Despite extensive stochastic exploration (100 total hops with a temperature parameter of 0.2), the basin‐hopping approach frequently exhibited higher loss distributions than the proposed framework across all eight batches (Figure 3). The advantage of the proposed framework is particularly evident in the correction of complex metabolite trajectories. For states exhibiting consumption‐production shifts, such as ammonium and lactate, the initial optimization frequently converged to high‐error local minima. While the basin‐hopping approach often struggled to resolve these specific trajectories, following LLM‐guided boundary updates, ammonium shows sustained loss reductions across most batches, while lactate exhibits notable improvements in Batches 4, 5, 6, and 8. These results demonstrate that the LLM‐guided framework improves the representation of these complex, nonlinear dynamics, highlighting the advantage of structure‐aware optimization over unguided stochastic searches. A detailed batch‐wise summary is provided in Section S5 of the Supporting Information (Table S2).

**Figure 3:**
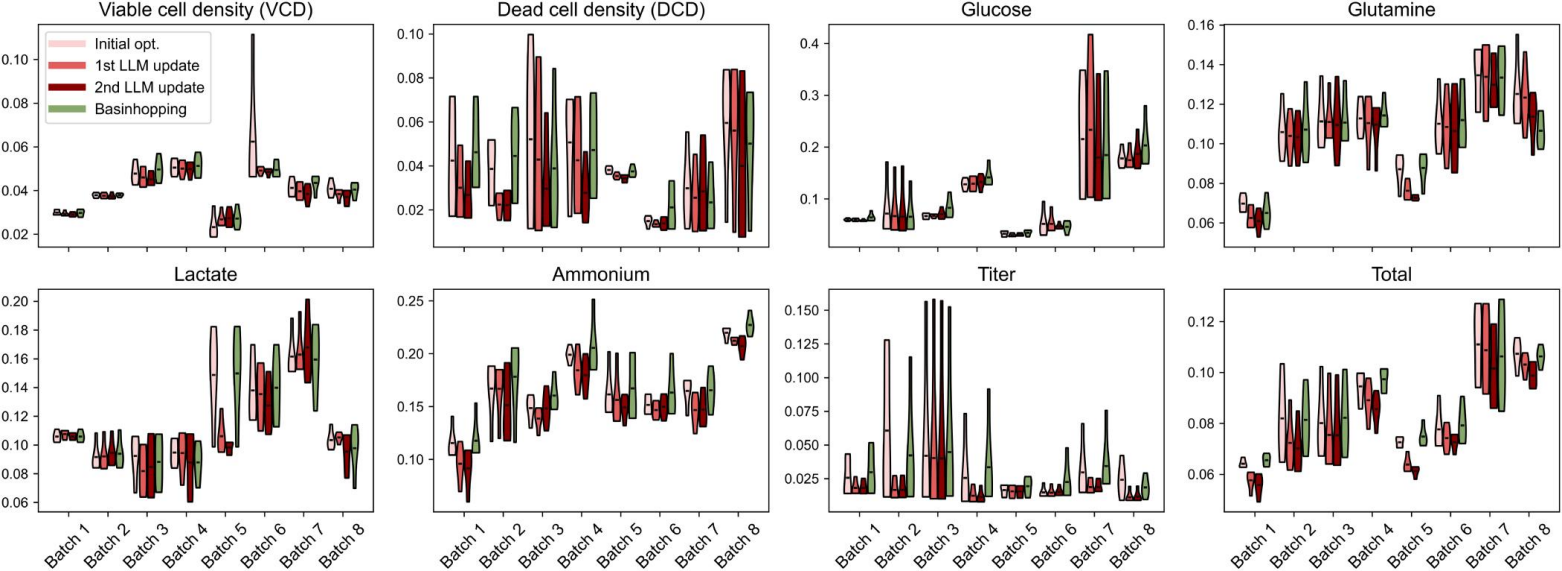
Statistical validation of the LLM‐guided parameter optimization against stochastic variability and an unguided global search algorithm. Objective loss distributions obtained from five independent random seed evaluations are shown for each of the eight batches, where “Total” denotes the unweighted mean of the min–max normalized MAEs across all states. For each batch, four violin plots represent the distributions corresponding to the initial optimization (lightest red), the first LLM‐guided (medium red), the second LLM‐guided update (darkest red), and the basin‐hopping approach (green). Across batches, the distributions progressively shift toward lower loss values following LLM‐guided updates, indicating consistent improvement beyond stochastic variation and the basin‐hopping optimization approach.

## 4 Conclusion

The integration of a LLM into the parameter estimation pipeline provides a systematic framework for embedding mechanistic reasoning into bioprocess modeling. As demonstrated, the proposed framework efficiently navigates high‐dimensional parameter spaces and enables consistent escape from local minima that typically trap conventional unguided solvers, particularly in capturing complex, nonlinear metabolic behaviors such as lactate and ammonium dynamics. Beyond improved convergence, the central contribution of this work lies in the automation of expert intuition within the optimization loop. By translating mechanistic understanding of governing equations into actionable boundary updates, the framework addresses a critical bottleneck in model calibration and enhances the robustness and reliability of model‐based design and real‐time process control within digital twin applications.

While the current framework demonstrates the promise of LLM‐guided optimization, several opportunities remain for further advancement. Incorporating richer mathematical and statistical contexts—such as parameter correlation structures and sensitivity information—could improve the consistency and physical interpretability of LLM‐driven updates. Additionally, computational efficiency may be further enhanced through the integration of more advanced convergence strategies and hybrid optimization schemes beyond the current stochastic global search framework. At the system level, replacing predefined phase segmentation with autonomous, data‐driven phase detection would enable more robust handling of complex metabolic transitions. Ultimately, evolving this approach into a fully integrated and self‐adaptive framework will facilitate broader and more reliable deployment of mechanistic digital twins across biomanufacturing processes.

## Author Contributions

**Han Bit Kim:** conceptualization, methodology, formal analysis, investigation, writing – original draft, visualization. **Janghan Lee:** resources, data curation. **Seo‐Yeon Kim:** resources, data curation. **Jihoon Kim:** resources, data curation. **Moo Sun Hong:** conceptualization, supervision, funding acquisition, project administration, writing – review and editing.

## Conflicts of Interest

The authors declare no conflicts of interest.

## Supporting information

Supporting File

## Data Availability

Experimental data sets are confidential.
