# Supplementary material for: LLM‐Guided Parameter Optimization for Mechanistic CHO Cell Bioreactor Models
Source: Biotechnol Bioeng. 2026 Jul 1;123(11):3175–82. doi: 10.1002/bit.70300 (PMC13665592; doi:10.1002/bit.70300)
Supplement: Supplementary file 1 [file BIT-123-3175-s001.pdf]

Supporting Information for

LLM-Guided Parameter Optimization for Mechanistic CHO Bioreactor Models

Han Bit Kim[1], Janghan Lee[2,3], Seo-Yeon KIM[2,3], Jihoon Kim[2], Moo Sun Hong[1,*]

[1]Department of Chemical and Biological Engineering, Seoul National University, Seoul 08826, Republic of Korea

[2]New Drug Development Center, Osong Medical Innovation Foundation, Cheongju 28160, Republic of Korea

[3]College of Pharmacy, Chungbuk National University, Cheongju 28160, Republic of Korea

[*]Corresponding Author: moosun@snu.ac.kr

Contents

S1 Complete kinetic model equations **2**

S2 Full text of the mechanistic structure-aware LLM prompt **3**

S3 Efficacy of iterative boundary contraction in global optimization **4**

S4 Full reasoning of the LLM agent for ammonium dynamics **5**

S5 Batch-wise summary of LLM-guided parameter boundary updates **6**

S6 References **7**

S1 Complete kinetic model equations

The complete set of ordinary differential equations (ODEs) describing the detailed metabolic kinetics, cell growth, death, and product formation is summarized below. As noted in the main text, these equations build upon the foundational model by Craven et al., with critical structural modifications including a dead cell lysis term and a self-limiting glutamine production term.

$$\frac{dX_\mathrm{v}}{dt} = (\mu - \mu_\mathrm{d})X_\mathrm{v} \tag{S1}$$

$$\frac{dX_\mathrm{d}}{dt} = \mu_\mathrm{d}X_\mathrm{v} - K_\mathrm{LYSIS}X_\mathrm{d} \tag{S2}$$

$$\mu = \mu_\mathrm{g,max}\left(\frac{\mathrm{Glc}}{K_\mathrm{G} + \mathrm{Glc}}\right)\left(\frac{\mathrm{Gln}}{K_\mathrm{Q} + \mathrm{Gln}}\right)\left(\frac{K_\mathrm{L}}{K_\mathrm{L} + \mathrm{Lac}}\right)\left(\frac{K_\mathrm{A}}{K_\mathrm{A} + \mathrm{Amm}}\right) \tag{S3}$$

$$\mu_\mathrm{d} = k_\mathrm{d,max}\left(\frac{\mathrm{Lac}}{KD_\mathrm{L} + \mathrm{Lac}}\right)\left(\frac{\mathrm{Amm}}{KD_\mathrm{A} + \mathrm{Amm}}\right) \tag{S4}$$

$$\frac{d\mathrm{Glc}}{dt} = -\left(\frac{\mu}{Y_{X/G}} + m_\mathrm{G}\right)X_\mathrm{v} \tag{S5}$$

$$\frac{d\mathrm{Gln}}{dt} = -\left(\frac{\mu}{Y_{X/Q}} + m_\mathrm{Q}\right)X_\mathrm{v} + k_\mathrm{Gln,prod}\frac{K_\mathrm{Gln,prod}}{K_\mathrm{Gln,prod} + \mathrm{Gln}}X_\mathrm{v} - k_{\mathrm{d_Q}}\mathrm{Gln} \tag{S6}$$

$$\frac{d\mathrm{Lac}}{dt} = Y_\mathrm{L/G}\left(\frac{\mu}{Y_\mathrm{X/G}} + m_\mathrm{G}\right)X_\mathrm{v} - k_\mathrm{Lac,cons}\frac{\mathrm{Lac}}{K_\mathrm{Lac,cons} + \mathrm{Lac}}X_\mathrm{v} \tag{S7}$$

$$\frac{d\mathrm{Amm}}{dt} = Y_\mathrm{A/Q}\left(\frac{\mu}{Y_\mathrm{X/Q}} + m_\mathrm{Q}\right)X_\mathrm{v} - k_\mathrm{Amm,cons}\frac{\mathrm{Amm}}{K_\mathrm{Amm,cons} + \mathrm{Amm}}X_\mathrm{v} + k_{\mathrm{d_Q}}\mathrm{Gln} \tag{S8}$$

$$\frac{dP}{dt} = (q_\mathrm{prod,1} + q_\mathrm{prod,2}\mu)X_\mathrm{v} \tag{S9}$$

State Variables: The key state variables and their respective units utilized in the model are defined as follows:

- X_v: Viable cell density (10^8 cells/L)
- X_d: Dead cell density (10^8 cells/L)
- Glc: Glucose concentration (mM)
- Gln: Glutamine concentration (mM)
- Lac: Lactate concentration (mM)
- Amm: Ammonium concentration (mM)
- P: Monoclonal antibody titer (mg/L)

Model Parameters: The default values for the 28 kinetic parameters utilized in the mechanistic model were established based on previously reported literature values (Amribt et al., 2013; Ben Yahia et al., 2015, 2021; Craven et al., 2013; García Münzer et al., 2015; Okamura et al., 2024; Xing et al., 2010) and are summarized in Table S1.

Table S1: Default values for the kinetic parameters of the CHO cell culture model.

**Parameter**	$\mu_\mathrm{g,max}$	$k_\mathrm{d,max}$	K_LYSIS	K_G	K_Q	K_L	K_A	KD_L	KD_A	$Y_\mathrm{X/G}$
**Default**	1.26	0.377	0.01	1.00	0.10	9.11	30.0	5.00	50.0	1.00
**Parameter**	$Y_\mathrm{X/Q}$	$Y_\mathrm{L/G}$	$Y_\mathrm{A/Q}$	m_G	m_Q	$k_\mathrm{Gln,prod}$	$k_\mathrm{Lac,cons}$	$k_\mathrm{Amm,cons}$	$K_\mathrm{Gln,prod}$	$K_\mathrm{Lac,cons}$
**Default**	1.0	1.0	1.0	10^{-6}	10^{-6}	0.1	0.1	0.1	0.1	10
**Parameter**	$K_\mathrm{Amm,cons}$	$k_{\mathrm{d_Q}}$	$k_\mathrm{Lac,prod}$	$K_\mathrm{Lac,prod}$	$k_\mathrm{Amm,prod}$	$K_\mathrm{Amm,prod}$	$q_\mathrm{prod,1}$	$q_\mathrm{prod,2}$		
**Default**	10	0.01	0.1	10	0.1	10	0.1	0.1		

S2 Full text of the mechanistic structure-aware LLM prompt

The following is the exact raw text of the prompt template provided to the LLM agent for directional boundary adjustments. Words enclosed in curly brackets (e.g., {model_equations_source}) denote dynamic variables that are programmatically injected at each iteration.

```
You are an expert in bioprocess modeling. Your task is to propose NEXT parameter *boundary*
    adjustments so the mechanistic CHO-cell reactor better matches measurements.

Inputs:
1) Mechanistic model equations
{model_equations_source}

2) Parameter descriptions
{params_block}

3) Trajectories:
{ts_block}

4) TARGET_STATE: {target_state_name}

Task:
Recommend NEXT parameter boundary changes to reduce the loss of TARGET_STATE.
- Select ONLY parameters that directly influence TARGET_STATE's ODE term(s): its production and
     consumption rates, and (when relevant) growth/death terms (\mu, \mu_d) that drive the
    state via r_* terms.
- You MUST use parameter names that literally appear in the equations above (use the exact
    spelling shown).
- Do NOT propose changes for unrelated states.
- Decisions allowed: 'up', or 'down'.
- Limit the number of recommended parameters to at most {max_params}.
Lever rules:
   **For creating convex/concave curves (curved shapes)**:
    - Purpose: Create curved, non-linear response patterns that match complex label shapes
    - Method: Increase BOTH production rates AND consumption rates simultaneously
    - Fine-tune half-saturation constants (K_*) or inhibition constants to control the balance
        between production and consumption
    - This allows both processes to compete dynamically, creating the desired curvature
   **For creating linear-like curves (straight shapes)**:
    - Purpose: Create straight, linear response patterns that match simple label trends
    - Method: Based on label trend direction, minimize either production OR consumption (not
        both)
    - If label is increasing: minimize consumption, maintain production
    - If label is decreasing: minimize production, maintain consumption
    - This creates linear response by allowing only one dominant process
```

S3 Efficacy of iterative boundary contraction in global optimization

To establish a robust baseline for our optimization pipeline, we validated the operational logic of the progressive parameter space refinement prior to LLM intervention. First, we conducted a comparative analysis to evaluate the efficacy of iterative boundary contraction against static wide-bound searches. As shown in Figure S1a, when distributing a fixed computational budget across multiple global iterations with progressively narrower bounds, the algorithm effectively stabilized and minimized the objective loss. This iterative strategy demonstrated significantly higher efficiency in navigating the high-dimensional parameter space and mitigating the risk of persistent local minima compared to a single evaluation with wide static boundaries.

Furthermore, analyzing the convergence trajectory of the global search justified our implementation of an early stopping criterion. As illustrated in Figure S1b, the objective loss systematically decreased as the search space was iteratively refined; however, the marginal improvement severely diminished as the number of global iterations approached 30 to 40. Consequently, we confirmed that terminating the global search when the objective function fails to exhibit meaningful improvement over 10 consecutive iterations successfully optimizes computational cost without compromising model accuracy.

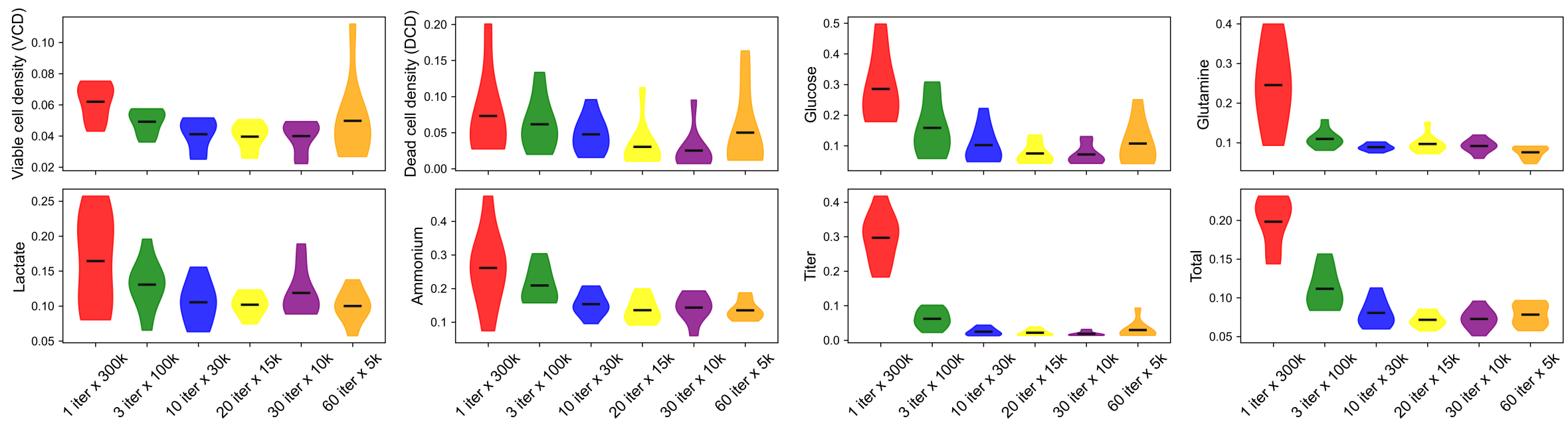

(a)

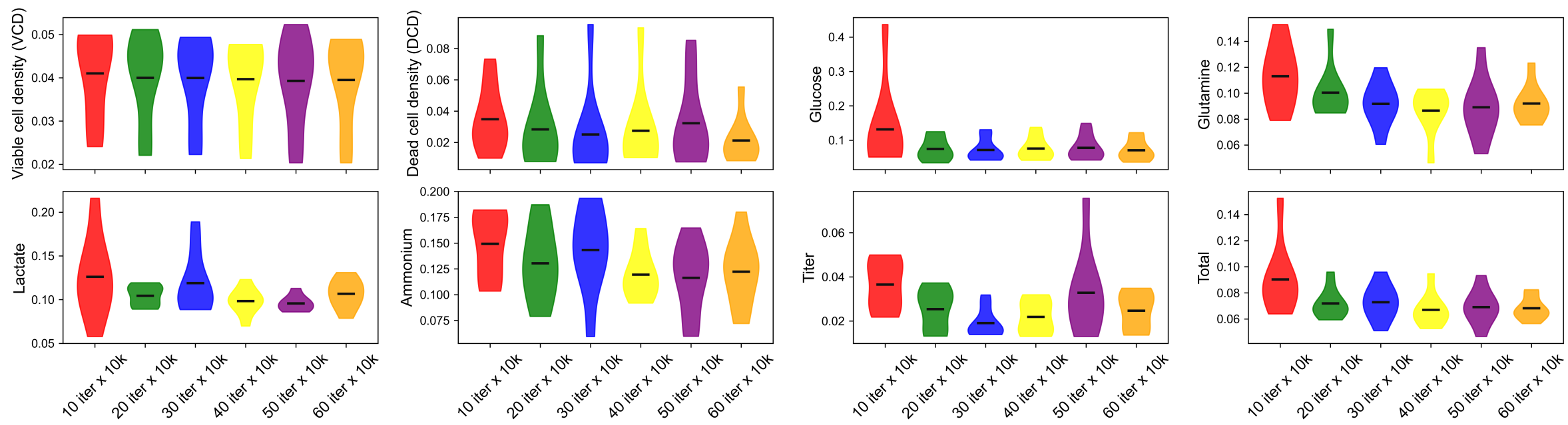

(b)

Figure S1: **Validation of the global search algorithm via progressive parameter space refinement.** (a) Comparison of optimization loss distributions under a fixed total computational budget ($n_{global\_iter} \times n_{exp\_per\_iter}$ = 300,000). A static wide-bound search (n_{global_iter} = 1, $n_{exp\_per\_iter}$ = 300,000) is compared against various iterative, boundary-contracting search strategies (e.g., n_{global_iter} = 30, $n_{exp\_per\_iter}$ = 10,000). (b) Convergence trajectory of the objective loss across increasing global iterations (n_{global_iter}), with a fixed evaluation budget per iteration ($n_{exp\_per\_iter}$ = 10,000).

S4 Full reasoning of the LLM agent for ammonium dynamics

The following text is the complete, unedited reasoning output generated by the LLM agent to rectify the initial ammonium trajectory, demonstrating its ability to leverage explicit mechanistic equations to formulate directional parameter updates.

Ammonium dynamics are governed by production:

$$r_{\text{amm,prod}} = Y_{\text{A/Q}} \cdot r_{\text{gln,cons}} + k_{\text{Amm,prod}} \cdot \frac{K_{\text{Amm,prod}}}{K_{\text{Amm,prod}} + \text{Amm}} \cdot X_{\text{v}}$$

and consumption:

$$r_{\text{amm,cons}} = k_{\text{Amm,cons}} \cdot \frac{\text{Amm}}{K_{\text{Amm,cons}} + \text{Amm}} \cdot X_{\text{v}}$$

plus a small contribution from glutamine degradation ($k_{\text{d,Q}} \cdot \text{Gln}$*).*

The measured ammonium profile shows a pronounced early peak that the model under-predicts, followed by a rapid decline to near-zero that the model over-predicts. To raise the early peak we increase the overall production capacity (raise $Y_{\text{A/Q}}$*, raise* $k_{\text{Amm,prod}}$*, raise* K_{A} *to lessen ammonium inhibition of growth and thus boost* $r_{\text{gln,cons}}$*). To sharpen the later decline we increase the consumption capacity (raise* $k_{\text{Amm,cons}}$*) and make the consumption term less saturated (lower* $K_{\text{Amm,cons}}$*).*

*This combination follows the **"curved-shape"** lever rule: simultaneously strengthening production and consumption while tuning half-saturation constants to allow the two processes to compete dynamically, reproducing the observed transient ammonium spike and subsequent clearance.*

S5 Batch-wise summary of LLM-guided parameter boundary updates

Table S2. Batch-wise summary of LLM-guided parameter boundary updates and corresponding loss reductions. The reported results correspond to the first metabolic phase (days 0–7.6), specifically evaluated using one representative random seed. For each batch, the target state, adjusted parameters with their directional guidance (↑: Up, ↓: Down), and the resulting Total loss (mean of normalized MAE across all states) are summarized for the first and second updates.

**Batch**	**Update**	**Target State**	**Adjusted Parameters & Direction**	**Total Loss (Before → After)**
Batch 1	1st	Ammonium	$Y_{A/Q}$ (↑), $k_{Amm,prod}$ (↑), $k_{Amm,cons}$ (↑), $K_{Amm,prod}$ (↑), $K_{Amm,cons}$ (↑)	0.0667 → 0.0608
	2nd	Lactate	$Y_{L/G}$ (↑), $k_{Lac,prod}$ (↑), $K_{Lac,prod}$ (↑), $k_{Lac,cons}$ (↑), $K_{Lac,cons}$ (↓)	0.0608 → 0.0601
Batch 2	1st	Ammonium	$k_{Amm,prod}$ (↓), $K_{Amm,prod}$ (↑), $k_{d,Q}$ (↓), $k_{Amm,cons}$ (↑), $K_{Amm,cons}$ (↓)	0.0772 → 0.0704
	2nd	Ammonium	$k_{Amm,prod}$ (↑), $K_{Amm,prod}$ (↑), $k_{Amm,cons}$ (↑), $K_{Amm,cons}$ (↓), $Y_{A/Q}$ (↑)	0.0704 → 0.0634
Batch 3	1st	Ammonium	$Y_{A/Q}$ (↑), $k_{Amm,prod}$ (↑), $k_{Amm,cons}$ (↑), $k_{d,Q}$ (↑), K_A (↑)	0.0672 → 0.0641
	2nd	Ammonium	$Y_{A/Q}$ (↑), $k_{Amm,prod}$ (↑), $K_{Amm,prod}$ (↑), $k_{Amm,cons}$ (↑), $K_{Amm,cons}$ (↓)	0.0641 → 0.0649
Batch 4	1st	Ammonium	$k_{Amm,prod}$ (↑), $k_{Amm,cons}$ (↑), $K_{Amm,cons}$ (↓), $Y_{A/Q}$ (↑), K_A (↑)	0.0972 → 0.0918
	2nd	Ammonium	$Y_{A/Q}$ (↓), $k_{Amm,prod}$ (↓), $K_{Amm,prod}$ (↓), $k_{d,Q}$ (↓), $k_{Amm,cons}$ (↑)	0.0918 → 0.0867
Batch 5	1st	Lactate	$k_{Lac,prod}$ (↑), $k_{Lac,cons}$ (↑), $K_{Lac,prod}$ (↑), $K_{Lac,cons}$ (↑), $Y_{L/G}$ (↑)	0.0744 → 0.0613
	2nd	Ammonium	$k_{Amm,prod}$ (↓), $K_{Amm,prod}$ (↓), $k_{Amm,cons}$ (↑), $K_{Amm,cons}$ (↓), $k_{d,Q}$ (↓)	0.0613 → 0.0607
Batch 6	1st	Lactate	$k_{Lac,prod}$ (↑), $k_{Lac,cons}$ (↑), $K_{Lac,prod}$ (↑), $K_{Lac,cons}$ (↓), $Y_{L/G}$ (↑)	0.0794 → 0.0777
	2nd	Lactate	$Y_{L/G}$ (↑), $k_{Lac,prod}$ (↑), $K_{Lac,prod}$ (↑), $k_{Lac,cons}$ (↑), $K_{Lac,cons}$ (↓)	0.0777 → 0.0757
Batch 7	1st	Glucose	$Y_{X/G}$ (↑), K_G (↑), m_G (↓), K_L (↓), K_A (↓)	0.0976 → 0.0950
	2nd	Glucose	$\mu_{g,max}$ (↓), K_G (↑), $Y_{X/G}$ (↑), m_G (↓), K_Q (↑)	0.0950 → 0.0947
Batch 8	1st	Glucose	$Y_{X/G}$ (↑), K_G (↓), m_G (↑), $\mu_{g,max}$ (↓), K_Q (↑)	0.1105 → 0.1040
	2nd	Glucose	$Y_{X/G}$ (↑), m_G (↓), K_G (↑), $\mu_{g,max}$ (↓), K_L (↓)	0.1040 → 0.0988

S6 References

Amribt, Z., Niu, H., & Bogaerts, P. (2013). Macroscopic modelling of overflow metabolism and model based optimization of hybridoma cell fed-batch cultures. *Biochemical Engineering Journal*, *70*, 196–209. https://doi.org/10.1016/j.bej.2012.11.005

Ben Yahia, B., Malphettes, L., & Heinzle, E. (2015). Macroscopic modeling of mammalian cell growth and metabolism. *Applied Microbiology and Biotechnology*, *99*(17), 7009–7024. https://doi.org/10.1007/s00253-015-6743-6

Ben Yahia, B., Malphettes, L., & Heinzle, E. (2021). Predictive macroscopic modeling of cell growth, metabolism and monoclonal antibody production: Case study of a CHO fed-batch production. *Metabolic Engineering*, *66*, 204–216. https://doi.org/10.1016/j.ymben.2021.04.004

Craven, S., Shirsat, N., Whelan, J., & Glennon, B. (2013). Process model comparison and transferability across bioreactor scales and modes of operation for a mammalian cell bioprocess. *Biotechnology Progress*, *29*(1), 186–196. https://doi.org/10.1002/btpr.1664

García Münzer, D. G., Ivarsson, M., Usaku, C., Habicher, T., Soos, M., Morbidelli, M., Pistikopoulos, E. N., & Mantalaris, A. (2015). An unstructured model of metabolic and temperature dependent cell cycle arrest in hybridoma batch and fed-batch cultures. *Biochemical Engineering Journal*, *93*, 260–273. https://doi.org/10.1016/j.bej.2014.10.013

Okamura, K., Badr, S., Ichida, Y., Yamada, A., & Sugiyama, H. (2024). Modeling of cell cultivation for monoclonal antibody production processes considering lactate metabolic shifts. *Biotechnology Progress*, *40*(6), e3486. https://doi.org/10.1002/btpr.3486

Xing, Z., Bishop, N., Leister, K., & Li, Z. J. (2010). Modeling kinetics of a large-scale fed-batch CHO cell culture by Markov chain Monte Carlo method. *Biotechnology Progress*, *26*(1), 208–219. https://doi.org/10.1002/btpr.284
